# Activity of Tracheal Cytotoxin of *Bordetella pertussis* in a Human Tracheobronchial 3D Tissue Model

**DOI:** 10.3389/fcimb.2020.614994

**Published:** 2021-01-19

**Authors:** David K. Kessie, Nina Lodes, Heike Oberwinkler, William E. Goldman, Thorsten Walles, Maria Steinke, Roy Gross

**Affiliations:** ^1^ Biocentre, Chair of Microbiology, University of Würzburg, Würzburg, Germany; ^2^ Chair of Tissue Engineering and Regenerative Medicine, University Hospital Würzburg, Würzburg, Germany; ^3^ Department of Microbiology and Immunology, University of North Carolina School of Medicine, Chapel Hill, NC, United States; ^4^ Department of Thoracic Surgery, University of Medicine Magdeburg, Magdeburg, Germany

**Keywords:** *Bordetella pertussis*, tracheal cytotoxin, airway epithelia, tissue model, ciliostasis, tight junction

## Abstract

*Bordetella pertussis* is a highly contagious pathogen which causes whooping cough in humans. A major pathophysiology of infection is the extrusion of ciliated cells and subsequent disruption of the respiratory mucosa. Tracheal cytotoxin (TCT) is the only virulence factor produced by *B. pertussis* that has been able to recapitulate this pathology in animal models. This pathophysiology is well characterized in a hamster tracheal model, but human data are lacking due to scarcity of donor material. We assessed the impact of TCT and lipopolysaccharide (LPS) on the functional integrity of the human airway mucosa by using *in vitro* airway mucosa models developed by co-culturing human tracheobronchial epithelial cells and human tracheobronchial fibroblasts on porcine small intestinal submucosa scaffold under airlift conditions. TCT and LPS either alone and in combination induced blebbing and necrosis of the ciliated epithelia. TCT and LPS induced loss of ciliated epithelial cells and hyper-mucus production which interfered with mucociliary clearance. In addition, the toxins had a disruptive effect on the tight junction organization, significantly reduced transepithelial electrical resistance and increased FITC-Dextran permeability after toxin incubation. In summary, the results indicate that TCT collaborates with LPS to induce the disruption of the human airway mucosa as reported for the hamster tracheal model.

## Introduction

*Bordetella pertussis* is a gram-negative bacterium that causes whooping cough mainly in humans. Children are the most susceptible although infections in the adult population is increasingly recognized (Guris et al., 1999; Durrheim et al., 2006; Galanis et al., 2006; Rohani et al., 2010). Pertussis is a highly contagious respiratory infection which is characterized by paroxysmal coughing fits with a distinctive “whoop” and post-tussive vomiting (Kilgore et al., 2016; Wu et al., 2019). Bacteria-containing aerosols generated during severe coughing episodes are considered to be the primary mode of disease transmission resulting in the colonization of the ciliated respiratory tract (Brockmeier & Lager, 2002; Warfel et al., 2012). Vaccines are readily available and widely applied, however, the disease persists worldwide with reported increases in incidence in some regions. Still, there are an estimated 2.4 million cases with about 640,000 deaths worldwide per year (Yeung et al., 2017). *B. pertussis* produces several virulence factors known to modulate host defense mechanisms required for an efficient colonization of the airway mucosa. These factors include several adhesins and toxins (Weiss et al., 1984; Anderton et al., 2004; Kilgore et al., 2016). Among the toxins the tracheal cytotoxin (TCT) stands out since it is a low molecular weight compound derived from the bacterial cell wall. The airway epithelium forms the first line of defense against invading pathogens. The mucociliary clearance mechanism and secreted mucins coupled with antimicrobial peptides such as β-defensins serve to inhibit bacterial colonization of the airway tissue. Furthermore, the epithelial cells possess various pattern recognition receptors which are able to sense peptidoglycan fragments from colonizing bacteria (Kvarnhammar et al., 2011; Wiese et al., 2017) possibly mounting an inflammatory response. As shown for the close relative *Bordetella bronchiseptica* in a canine model, infection leads to the dysfunction of the mucociliary clearance mechanism and mucosa damage allowing the bacteria to colonize the epithelium (Anderton et al., 2004). TCT has been implicated in the destruction of the ciliated epithelia of the airway in animal models and in human nasal biopsies (Goldman & Cookson, 1988; Wilson et al., 1991; Luker et al., 1993), thus hindering the mucociliary clearance mechanism and indicating that TCT may be a potentially important virulence factor of *B. pertussis*.

TCT is a 921-Da muramyl peptide released at the logarithmic growth phase during peptidoglycan cell wall turnover (Goldman & Cookson, 1988). It is common to all gram-negative bacteria but usually it is efficiently recycled by the AmpG permease into the cytoplasm (Goodell, 1985; Park, 2001; Uehara & Park, 2004). However, in *Bordetella pertussis*, the AmpG permease is defective leading to an accumulation of the toxin in the periplasm and, by an unknown mechanism, to its release into the supernatant (Cookson et al., 1989). TCT is also reported to be released by *Vibrio fischeri* causing important tissue arrangement required for the successful formation of the symbiotic light organ in Hawaiian bobtail squids (Koropatnick et al., 2004). Additionally, *Neisseria gonorrhoeae* releases peptidoglycan fragments including TCT into the culture supernatant which have been shown to induce similar damage to the ciliated epithelium of the fallopian tube (Melly et al., 1984) as reported for *B. pertussis* in the trachea, although the underlying mechanisms are poorly understood (Chan & Dillard, 2016). TCT was reported to provoke the expression of the inducible nitric oxide synthase (iNOS) in hamster tracheal epithelial cells and explants in synergy with lipopolysaccharide (LPS) (Flak & Goldman, 1996; Flak & Goldman, 1999; Flak et al., 2000). It was proposed that this subsequently leads to an increased production of nitric oxide (NO) to cytotoxic levels which may contribute to the characteristic cytopathology of pertussis, i.e., the destruction of the ciliated epithelial layer. Paik et al. (2017) reported that overexpression of the solute carrier family 46 member 2 (SLC46A2), a member of the thymic stromal cotransporters, in HEK293 cells resulted in a higher sensitivity of the cells to TCT and upregulation of NOD1 mediated NF-κβ expression. This indicates a role of SLC46A2 in the transport of TCT into the cytoplasm of the host cell enabling its interaction with NOD1. However, the role of TCT induced NO production in the disruption of the human airway mucosa so far remains unclear since relevant human models were lacking, but also because TCT induced NO concentrations may not reach toxic levels due to volatility in the strongly ventilated airways. Nevertheless, for safety reasons in attempts to develop a new live attenuated pertussis vaccine, the respective strain BPZE1 was engineered in a way to lack TCT secretion (Locht et al., 2017).

The advancement of tissue engineering approaches provides an opportunity to develop complex tissue and organ models using primary human cells to address some of these issues. Due to their high *in vivo-in vitro* correlation, these 3D models are valuable tools to study bacteria-host interactions without the limitations associated with flat cultures and animal models. Previously we developed a 3D airway mucosa model using primary human tracheobronchial epithelial cells and dermal fibroblasts cultured on a biological scaffold (Steinke et al., 2014). Infection of these tissue models with virulent *B. pertussis* and an avirulent mutant (unpublished data) not expressing the known virulence proteins resulted in mucosa disruption and necrosis, suggesting that TCT, the only known toxin produced by the avirulent mutant, may be responsible for the observed tissue damage.

In this study, we used airway mucosa models developed from human tracheobronchial epithelial cells and fibroblasts to assess the effect of purified TCT and LPS. We show that TCT alone is able to induce blebbing and necrosis of airway epithelial cells. Massive blebbing of epithelial cells leads to loss of ciliated cells and thus to impairment of mucociliary clearance. Preliminary data suggest that a TCT dependent NO production and pro-inflammatory cytokine expression correlated with TCT induced tissue damage.

## Materials and Methods

### Donor Tissue and Primary Cells

Tracheobronchial epithelial cells and fibroblasts were isolated from bronchial biopsies surgically resected from 5 donors (aged 45–70) undergoing selective bronchial/lung resections at the Department of Thoracic Surgery, Otto-von-Guericke University Magdeburg, Germany. Informed consent was obtained from patients before surgery and the studies were approved by the institutional ethics committees on human research of the Otto-von-Guericke University Magdeburg (vote 163/17) and Julius-Maximilians-University Würzburg (vote 179/17), respectively.

### Cell Culture and Generation of 3D Tracheobronchial Mucosa Tissue Models

Primary tracheobronchial epithelial cells were isolated from mechanically removed airway mucosa from tracheobronchial biopsies as previously described by Steinke et al. (2014). Fibroblasts were isolated from the same tissue specimen as described in Schweinlin et al. (2017). The preparation of the porcine small intestine submucosa (SIS) scaffold was done as described by Schweinlin et al. (2016). The tracheobronchial epithelia cells were isolated and maintained in serum-free human Airway Epithelial Cell growth medium [(AECGM), #PB-(C)-MH-350-0099, PELOBiotech, Germany] at 37°C and 5% CO_2_ in a humidified incubator until 80% to 90% confluent. Tracheobronchial fibroblasts were isolated and maintained in fibroblast growth medium (FGM) consisting of Dulbecco’s Modified Eagles Medium (Thermo Fisher, Germany) supplemented with 10% fetal bovine serum. All media for cell culture contained 0.1 mg/ml Streptomycin, 100 U/ml penicillin and 0.25 µg/ml amphotericin B. The human tracheobronchial mucosa models (hTBM) were developed as previously described by Steinke et al. (2014) and Schweinlin et al. (2017) using the SIS scaffold. Briefly, pieces of the SIS scaffold were mounted on plastic 12 mm diameter cell crowns and incubated for 2 h with media consisting 1:1 mix of AECGM and FGM. 100,000 fibroblasts were seeded from the apical side of the cell crown and 300,000 tracheobronchial epithelial cells were seeded 24 h later on the apical side as well. The cells were cultured under submerged conditions for 3 days and subsequently under airlift conditions for 21–28 days. The hTBM were analyzed with a Dalsa motion traveler high-speed video camera (Imaging Solutions GmbH, Germany) mounted on a Nikon Eclipse 80i microscope (Nikon GmbH, Germany) and considered matured when beating cilia were observed usually after 21–28 days under airlift conditions. These models were used for the toxin experiments. Each experiment was performed in triplicates unless otherwise stated.

### Tracheal Cytotoxin Assay

The fully differentiated hTBM were washed 3x with fresh mixed medium (1:1 AECG: FGM) to wash away some of the mucus prior to incubation with the toxins. The hTBM were then treated with 300µl each of 3µM TCT, 100 ng/ml LPS (*Escherichia coli* 026:B6, Sigma Aldrich, USA) and a mixture of TCT and LPS (TCT/LPS) apically and incubated in a humidified incubator at 37°C and 5% CO_2_ for 24 h. *E. coli* LPS was chosen for this study in order to make the data directly comparable to the previously published hamster tracheal ring experiments in which *E. coli* LPS was also used (Flak & Goldman, 1999; Flak et al., 2000). A mock control was setup by adding 300µl of freshly prepared mixed media without any toxin to the apical compartment and 1 ml to the basal compartment. The basal compartment contained 1 ml of fresh medium for all the treatment groups. All the toxin experiments as well as the mock control was performed under submerged conditions. To determine the effect of ventilation, the hTBM were placed for 30 min under the flow hood with the well plate open. This was repeated after every 2 h incubation for 12 h. The supernatants from the apical and basal compartment were analyzed for NO production using the Griess test.

### Quantification of Nitric Oxide

Dissolved nitric oxide in the supernatant after 24 h of toxin incubation was quantified with Griess reagent (Promega #G2930, USA) and performed according to company’s instructions. Briefly, 100µl of the culture supernatant from the apical and basal compartments were mixed with 100µl of the Griess reagent and incubated for 15 min at room temperature. Absorbance was measured at 548 nm using a TECAN infinite M200 microplate reader (TECAN GmbH, Germany). The supernatants from 3 independent experiments were used to determine the amount of nitric oxide (NO) released upon toxin incubation.

### Determination of Ciliary Beat Frequency

The hTBM were transferred from the cell crowns onto silicon coated Delta T dishes (Bioptechs Inc, USA) and held in place with insect pinning needles (Ento Sphinx, Czech Republic). The tissue models were submerged in mixed media and the temperature was maintained at 37°C throughout the recording of the videos with a Bioptechs Delta T5 µ-Environmental culture dish controller (Bioptechs Inc, USA). Videos of beating cilia were recorded with a Nikon Eclipse 80i microscope equipped with a water immersion Achroplan 40×/0.80W objective (Nikon GmbH, Japan). At least 5 different areas of each model were selected, and movies of cilia motion was recorded at 100 Hz frame rate and 640 X 480 pixels prior to incubation with toxins and 24 h after toxin incubation. The mean ciliary beat frequency (CBF) was calculated from at least 10 randomly selected ciliated cells in each movie from 3 independent experiments. The CBF was determined by the Fourier transformation of grey level changes over time with a Mathlab (MathWorks, USA) script developed by Prof. Dr. Peter König from the University of Lübeck, Germany (Lindner et al., 2017).

### Assessment of Mucociliary Transport

The mucociliary transport apparatus of the hTBM was assessed using the particle transport assay. The hTBM were transferred from the cell crown onto silicon coated Delta T-dishes and fixed in place as described above. The models were then washed with prewarmed sterile PBS and covered with 1 ml fresh mixed medium. The temperature was maintained at 37°C throughout the recording of the videos with a Bioptechs Delta T5 µ-Environmental culture dish controller (Bioptechs Inc, USA). Dynabeads™ Protein G (Thermo Scientific, USA) were suspended in fresh mixed medium and added to a final concentration of 15 µg/ml. Using a 20×/0.5W water immersion objective, 15-s-long videos of the beads were recorded at 200fps before and 24 h after toxin treatment. The videos were then analyzed with Image-Pro Premier software (Media Cybernetics, USA) to determine the displacement of the beads. About 10 particles per treatment group from 3 independent experiments were tracked over 100 frames to determine their displacement.

### Immunofluorescence Staining and Confocal Microscopy

The tissue models were washed 3x with cold sterile Dulbecco’s phosphate buffered saline (DPBS) and fixed in 4% PFA for 2 h at room temperature on the cell crowns. The models were then washed with PBS, blocked and permeabilized with 5% bovine serum albumin (BSA) containing 0.01% Triton X-100 in PBS for 1 h at room temperature. The tissue models were incubated with the primary antibodies diluted with 3% BSA in PBS overnight at 4°C. The models were washed 3x with PBS containing 0.5% Tween-20 and counterstained with fluorophore-conjugated secondary antibodies at room temperature for 1 h. The hTBM were then mounted on glass slides with fluoromount-G with DAPI (Thermo Scientific, USA). A Z-stack of images was obtained through 25µm from 3 independent experiments with a LEICA SP8 Confocal Laser Scanning microscope (Leica Microsystems, Germany) and processed with FIJI (Schindelin et al., 2012).

### Antibodies

The primary antibodies used in this study are listed on **Supplementary Table 1**. The fluorophore conjugated secondary antibodies used were polyclonal donkey anti-mouse Alexa Fluor 488 (1:400, A21202, Thermo Scientific, USA) and polyclonal donkey anti-rabbit Alexa Fluor 647 (1:400, A31573, Thermo Scientific, USA).

### Electron Microscopy

Ultrastructural analysis of the hTBM after toxin treatment was performed at the Biocentre Imaging Core Facility of the University of Würzburg. The tissues were fixed in 2.5% cacodylate-buffered glutaraldehyde (50 mM cacodylate, pH 7.2, 50 mM KCl, and 2.5% MgCl_2_) at 4°C for transmission electron microscopy and in 6.5% glutaraldehyde for scanning electron microscopy. Further processing proceeded as previously described (Spiliotis et al., 2008) and the tissues were examined on the JEOL JEM-2100 TEM and JEOL JSM-7500F SEM (JEOL, Japan). The whole surface of the tissue from 3 different experiments was initially scanned at low magnification to observe the level of disruption after which high magnification images were made.

### Transepithelial Electrical Resistance and Fluorescein Isothiocyanate-Dextran Permeability

TEER was used to assess the barrier integrity of the human tracheobronchial mucosa models. The TEER was measured using Millicel^l®^ ERS-2 Volt-Ohm meter (Merck, Germany). The TEER of the airway models were measured before and 24 h post toxin addition. The barrier integrity was additionally assessed with a 4kDa fluorescein isothiocyanate-dextran (FITC-Dextran, Sigma, Germany) permeability assay before and after the 24-h period of toxin incubation. Briefly, 0.25 mg/ml of FITC-Dextran were dissolved in cell culture medium and sterile-filtered. The hTBM were washed with sterile PBS and 2 ml fresh mixed medium were added to the basal compartment. 500µl of medium containing FITC-Dextran was added to the apical compartment. 200µl of the media from the lower compartment were collected after 30 min incubation into 96 well plates and analyzed in a TECAN reader (absorbance, 490 nm; emission, 525 nm). The mean absorbance measured for three different experiments was normalized to a cell-free SIS scaffold mounted on a cell crown. The permeability of the hTBM to FITC-Dextran was expressed as a percentage after normalization with the cell-free SIS scaffold.

### RNA Isolation and Quantitative RT-PCR

The hTBM were lysed by agitating for 2 min at 50 rpm in lysis buffer containing β-Mercaptoethanol prior to RNA isolation at 4°C. Total RNA was isolated using the Qiagen RNA isolation kit (Qiagen, Germany) according to the manufacturer’s instruction. Isolated total-RNA quality and quantity was determined using TECAN infinite M200 microplate reader (TECAN GmbH, Germany). 1ug of the total RNA was transcribed to cDNA with iScript™ cDNA synthesis kit (Bio-Rad, USA). The cDNA was quantified with the SSOFast™ EvaGreen^®^ Supermix (Bio-Rad, USA) on the Sens Quest CFX96 real time PCR thermocycler (Bio-Rad, USA). The GAPDH gene was used as reference in all the quantitative RT-PCR experiments. Primers were designed using Primer3 (**Supplementary Table 2**) and were used at a final concentration of 500nM. A 3-cycle amplification method was performed with cycling conditions as follows: (i) initial denaturation at 95°C 3 min, followed by 40 cycles of (ii) denaturation at 95°C for 10 s, (iii) primer annealing at 65°C for 30 s and (iv) stabilization, elongation and fluorescence detection at 72°C for 30 s. The gene expression fold change was calculated using the 2^(-ΔΔCT)^ Livak threshold cycle (C_T_) method (Livak & Schmittgen, 2001)

### Cytokine Bead Assay

Fifty microliters of cell culture supernatants were collected from the apical and basal compartments of 24-h toxin treated hTBM into 1.5-ml Eppendorf tubes. The supernatants were centrifuged at 12,000 rpm at 4°C and the upper media transferred into fresh Eppendorf tubes. Samples were frozen at −80°C overnight. The cytokines in the supernatant were quantified using the BD human inflammatory cytokine Cytometric Bead Array (CBA) kit (cat. 551811, Becton, Dickinson and Company, USA) which allows the simultaneous quantification of up to 6 inflammatory cytokines (IL-1β, IL-6, IL-8, IL-10, TNF, IL-12p70) in 12.5 µl of sample volume. For this study, we quantified IL-1β, IL-6, IL-8 and IL-10 in the cell culture supernatant. The test was performed according to company instructions. The cytokine capture beads were assessed using a BD Accuri™ C6 Plus flow cytometer (Becton, Dickinson and Company, USA). The cytokines were quantified using the FCAP Array™ software version 3.0 (Becton, Dickinson and Company, USA). A 1:50 dilution of the supernatants was done to lower the IL-6 and IL-8 levels within the limits of detection of the kit. The inflammatory cytokine concentrations from the treatment groups were normalized to control concentration and expressed as relative concentrations to eliminate donor response variations. The concentration of the inflammatory cytokines in pg/ml is shown in **Supplementary Table 3**.

### Statistical Analysis

An ordinary one-way or two-way analysis of variance statistics was performed using GraphPad Prism version 8 (GraphPad Software Inc., USA) for all quantitative datasets. A Tukey post-hoc analysis was used for multiple comparisons. Statistical significance was tested at 95% confidence level (p<0.05). Data represent means of at least three independent experiments performed in duplicate.

## Results

### TCT Causes Cellular Blebbing and Extrusion in Airway Mucosa Tissue Models

**Figure 1:**
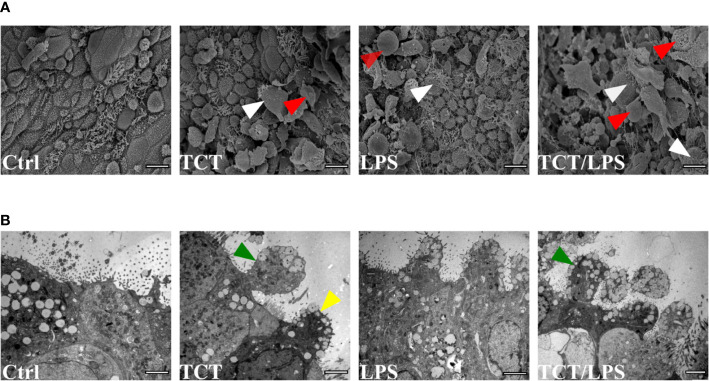
TCT and LPS induce the disruption of human airway mucosa. The hTBM were incubated with 300µl of 3µM TCT, 100ng/ml LPS and a combination of TCT/LPS for 24 h. Control hTBM were mock treated by incubating them submerged with 300µl fresh mixed medium in the apical compartment and 1ml in the basal compartment. **(A)** Scanning electron micrographs of hTBM after 24 h of toxin incubation. TCT and LPS in combination elicited a more severe damage compared to either toxins alone. Both ciliated (white arrowhead) and nonciliated cells (red arrowhead) were extruded from the hTBM. Scale bars: 10µm. **(B)** Transmission electron micrographs show blebbing of denuded ciliated cells (green arrowhead) and nonciliated goblet (yellow arrowhead) of hTBM after 24 h of toxin incubation. Scale bars: 2µm.

Previous studies by Goldman et al. (1982) using hamster tracheal explants and Wilson et al. (1991) using human nasal brush biopsies implicated TCT in the loss of ciliary cells by extrusion. To determine if TCT has the same effect on the human airway mucosa *in vitro*, we developed tracheobronchial mucosa models as described (Steinke et al., 2014; Schweinlin et al., 2017; Lodes et al., 2020). The human tracheobronchial mucosa models (hTBM) were characterized and confirmed to possess airway mucosa properties such as beta tubulin-positive ciliated cells, CK14-positive epithelial cells, CK18-positive differentiated epithelial cells, mucous producing goblet cells (Muc5AC- and Muc5B-positive) and CK5-positive basal cells as previously described (Jeffery and Li, 1997; Steinke et al., 2014; Xu et al., 2016; Lodes et al., 2020). Tracheal fibroblasts were immunostained for vimentin to show their location in the scaffold (**Figure S1**). We incubated the hTBM with 3µM TCT and/or 100 ng/ml LPS for 24 h from the apical side in order to mimic the direction of contact in a natural infection. To assess the impact of the toxins on the hTBM, the models were fixed, and the surface analyzed using scanning electron microscopy. The scanning electron micrographs showed extruded cells from the mucosa of the hTBM treated with TCT alone or LPS alone compared to controls (**Figure 1A**). The combination of TCT and LPS resulted in even more severe damage to the airway mucosa models (**Figure 1A**). The scanning electron micrographs also revealed that the surface of the hTBM incubated with a combination of TCT and LPS was largely covered with cell debris and extruded cells. Transmission electron micrographs show that the effect of the toxins was not restricted to ciliated cells alone. **Figure 1B** shows that both ciliated and non-ciliated cells such as goblet cells were extruded from the hTBM as well. While TCT alone and LPS alone were observed to cause pinching of part of the cells, a combination of the two toxins resulted in complete extrusion of cells from the airway mucosa models (**Figure 1B**).

### TCT/LPS Impairs Mucociliary Clearance

**Figure 2:**
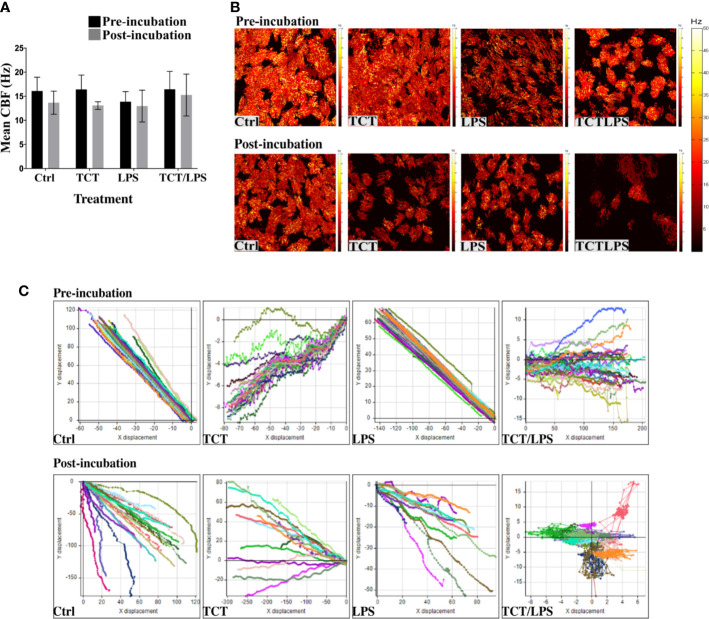
Tracheal cytotoxin does not significantly affect ciliary beat frequency (CBF). **(A)** Mean CBF of at least 5 movies recorded at 100fps and at least 10 ciliated cells of the hTBM were measured before toxin incubation and 24 h after toxin incubation. Bar graph shows mean ± SEM of three independent experiments. **(B)** Representative heat maps of ciliated cell distribution and ciliary beat frequency before and 24 h after toxin incubation. **(C)** Displacement of Dynabeads™ before and after treatment over 100 frames. The particle transport ability of the hTBM were assessed before and after toxin treatment. Control, TCT and LPS treated models retained the ability to displace the beads from their point of origin in both the x and y plane. The Dynabeads™ in TCT/LPS treated hTBM oscillated around their point of origin. The data shows displacement of tracked particles over 100 frames in 1s.

One feature of *B. pertussis* infection is the dysfunction of the mucociliary clearance mechanism which is characterized by a reduction and/or loss of ciliary activity. Previous reports suggested that TCT induced ciliostasis in hamster tracheal explants (Goldman & Cookson, 1988; Luker et al., 1993). To assay the potential of TCT to recapitulate this pathology *in vitro* in human specimens, ciliary beat frequency (CBF) of the models was assessed prior to and 24 h after toxin addition as described by Lindner et al. (2017). Surprisingly, TCT and LPS did not have an acute effect on the CBF either alone or in combination after 24 h. The mean CBF from all treatment groups remained above the minimum CBF of 9 Hz (Rutland et al., 1982; Dresdner & Wong, 1985; Katz et al., 1987) reported for normal human airway ciliated epithelia (**Figure 2A**). However, a careful evaluation of the models revealed the existence of some ciliated cells with non-beating cilia or with a strongly reduced movement in models treated with LPS (**Supplementary Video 6**) and with the combination of TCT and LPS (**Supplementary Video 8**). As shown in **Figure 2B**, a qualitative evaluation of the models incubated with the toxins overall revealed a lower amount of beating kinocilia as compared to control models and, in addition, an increased production of mucus upon intoxication (**Supplementary Video 1**-**8**). To assess the effect of the toxins on the mucociliary clearance mechanism, a particle transport assay using the displacement of Dynabeads™ Protein G was performed. As shown in **Supplementary Videos (9**–**11** **;** **13**–**15** **)**, incubation of the models did not affect the motility of Dynabeads™ in hTBM treated with TCT alone, LPS alone as well as controls. However, TCT/LPS treated hTBM were unable to displace the beads as they seemed to be trapped in the mucus despite the presence of functional cilia (**Supplementary Video 16**). Analysis of the displacement patterns with the Image Pro Premier software showed a change in the Y and X coordinates in the control, TCT and LPS treated hTBM before and after the 24-h treatment (**Figure 2C**, **Supplementary Figure 5**). The Dynabeads™ seemed to oscillate around the point of origin in TCT/LPS treated hTBM (**Figure 2C**). The observed differences in particle trajectories in **Figure 2C** (**Supplementary 5**) were due to the orientation of the models on the Delta T plates during recording of the videos. Taken together, our results indicate the combination of TCT and LPS may contribute to the impairment of mucociliary clearance *in vivo*.

### TCT Action Leads to Loss of Barrier Function of Airway Mucosa Models

**Figure 3:**
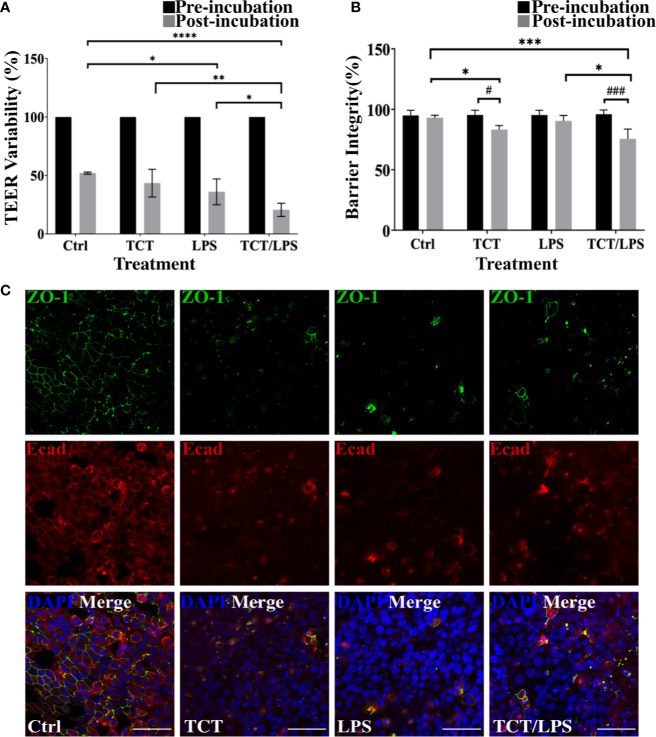
TCT incubation compromises barrier integrity of airway mucosa model. **(A)** the hTBM were incubated with 3µM TCT, 100ng/ml LPS, mixture of TCT/LPS or mock treated by adding fresh mixed media to the apical compartment for 24 h. The transepithelial electrical resistance for the models was measured prior to and 24 h after toxin addition. Only hTBM treated with a combination of TCT and LPS showed a significant difference in TEER compared to controls post incubation. The bar chart shows mean ± SEM of the TEER measured from duplicate experiments from 3 different donors (N=6) **(B)** The barrier integrity (%) of the airway mucosa models to 4 kDa FITC-Dextran was determined before and after 24 h toxin incubation. The data represent mean ± SEM of 3 independent experiments performed in duplicates (N=6). Statistical significance was tested with Tukey’s two-way ANOVA. * denotes statistical significance between treatment groups after the 24 h incubation period (*p≤0.05, **p≤0.01, ***p<0.005, ****p<0.001). # denotes statistical comparison before and after 24 h incubation within treatment groups (^#^p≤0.05, ^##^p≤0.01). **(C)** Representative hTBM showing the tight junction proteins ZO-1 (green) and E-cadherin (red) organization after 24 h incubation with TCT, LPS, and TCT/LPS. Scale bars: 50µm.

To assess the impact of toxin incubation on the airway mucosa models, the transepithelial electrical resistance (TEER) was measured prior to incubation of the toxins and 24 h after toxin incubation. The mean TEER of the untreated hTBM was 19.66±0.35 Ωcm^2^. In **Figure 3A** the changes in the TEER values after toxin treatments were normalized to the pre-treatment TEER and expressed as a percentage. As shown in **Figure 3A**, incubation of the airway mucosa model under submerged conditions for 24 h compromises the transepithelial electrical resistance of the airway mucosa model. In the mock treated control hTBM, a 48.06% decline in the TEER was observed after the 24-h incubation period. Thus, the incubation of the hTBM with time caused a reduction in their TEER values, but the presence of the toxins significantly boosted this effect. Treatment of the hTBM with either TCT or LPS resulted in 56.61% and 64.04% decline in the TEER respectively. The combination of TCT and LPS had an even stronger effect and significantly reduced the TEER by 79,44%. Compared to the mock treated hTBM, LPS alone (p=0.0139) and TCT/LPS (p<0.0001) showed statistically significant decline in TEER after 24 h (**Figure 3A**). TCT/LPS also induced significantly higher reduction in TEER compared to either toxin alone (**Figure 3A**).

To determine if the observed drop in TEER of the airway mucosa model corresponded to an increased permeability of the hTBM, we performed a FITC-Dextran permeability test. Interestingly, although the incubation of the hTBM submerged with media alone resulted in a drop in TEER, this reduction did not translate into increased permeability for FITC-Dextran. The permeability of the control treated hTBM for the FITC-Dextran normalized to a blanked SIS scaffold remained unchanged. The hTBM incubated with LPS alone showed a 10% increase in permeability to FITC-Dextran although it was not as significant compared to mock treated controls as suggested by the TEER (**Figure 3B**). Incubation with TCT alone resulted in a significant (p=0.0480) decline of the barrier integrity of the hTBM by 18%. The combination of TCT/LPS caused the most significant (p=0.0007) decrease of 27% in the barrier integrity after the 24-h incubation period (**Figure 3B**). Since barrier integrity and TEER of the airway mucosa models depend on tight junction organization, the hTBM were fixed with 4% PFA and the tight junction-associated proteins were visualized by double immunofluorescence staining using antibodies against ZO-1 and E-cadherin. Mock treated hTBM showed continuous labeling of ZO-1 and E-cadherin while the hTBM treated with TCT and/or LPS showed discontinuity in E-cadherin expression and complete disorganization of ZO-1(**Figure 3C**). Moreover, the hTBM incubated with the toxins displayed a clear loss of ZO-1 and E-cadherin organization after 24 h which could account for the observed decreases in the TEER values (**Figure 3C**).

### TCT Induces the Production of Nitric Oxide From Airway Mucosa

**Figure 4:**
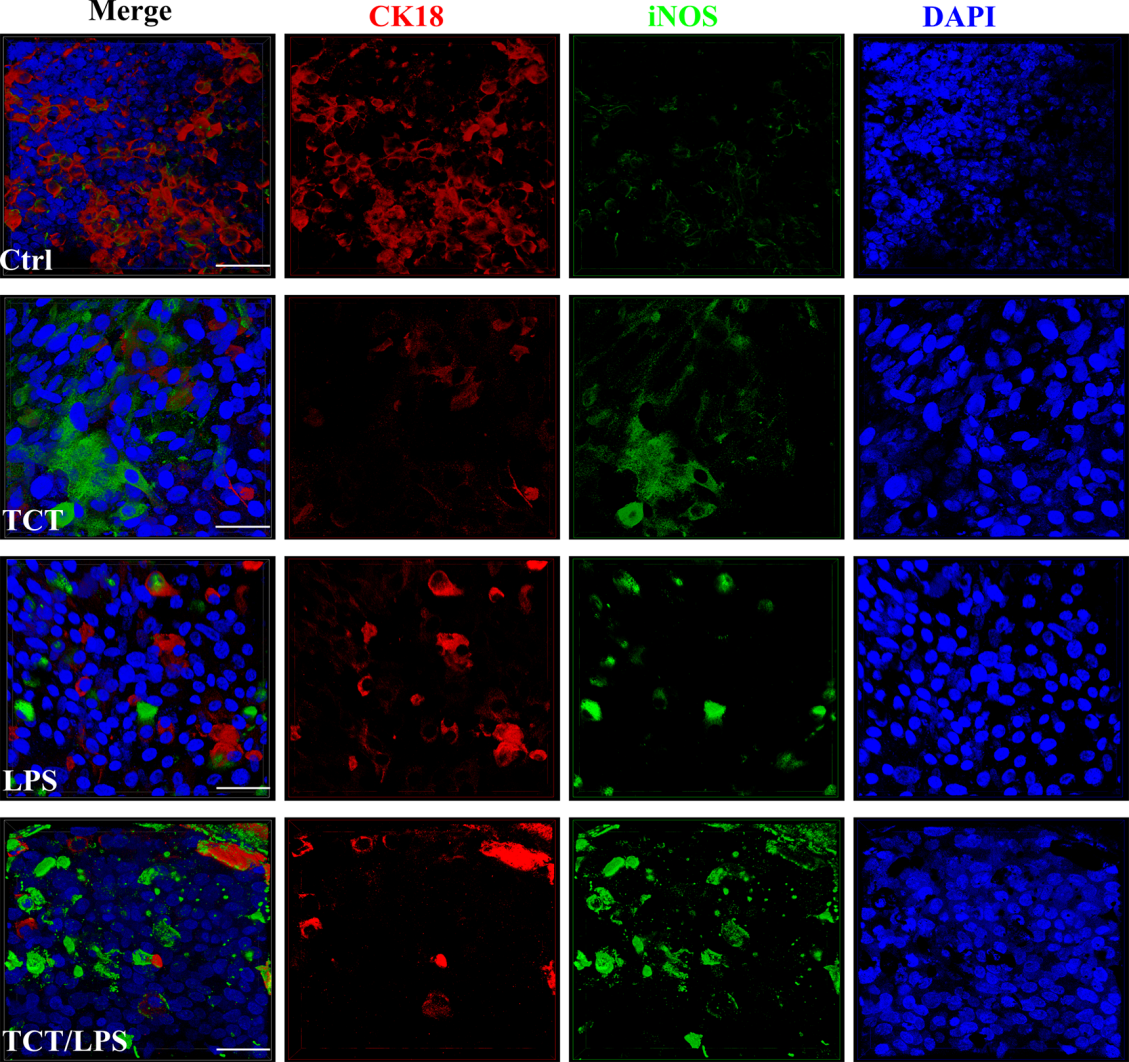
Secretion of NO and expression of iNOS after intoxication of the hTBM with TCT and LPS. The hTBM were treated with 3 µM TCT and/or 100 ng/ml LPS from the apical compartment. Immunofluorescent staining of the hTBM with an iNOS-specific antibody to visualize iNOS (green) induction after toxin treatment after 24 h of incubation. iNOS expression was directed towards the apical surface of the polarized airway epithelia. The epithelial cells were counterstained with CK18 (Red). Scale bars 50µm.

The ability of TCT to induce epithelial cell blebbing is reported to be mediated by the induction of toxic concentrations of nitric oxide in hamster tracheal explants and cells (Heiss et al., 1994; Flak and Goldman, 1996; Flak and Goldman, 1999; Flak et al., 2000). Thus, dissolved NO in the cell culture supernatants from the apical and basal compartments of the hTBM was measured using the Griess reagent 24 h after toxin addition. Compared to controls, TCT, LPS and the combination of TCT/LPS seemed to consistently induce an increased NO production, however, due to a strong variability among the different models generated with primary epithelial cells from different donors, the differences were statistically not significant (**Supplementary Figure 2A**). Ventilation of the models did not have a significant effect on the quantity of dissolved NO in the supernatants as shown in **Supplementary Figure 2B**. To determine if the apparent toxin mediated increase of released NO was due to iNOS, the hTBM were fixed with 4% PFA and decorated with an anti-iNOS specific antibody which does not cross-react with eNOS and cNOS. Confocal microscopy indicated that both TCT and LPS alone, as well as in combination induced an increase in the expression of iNOS compared to controls (**Figure 4**).

### TCT Elicits Inflammatory Cytokine Release From Airway Mucosa Models

**Figure 5:**
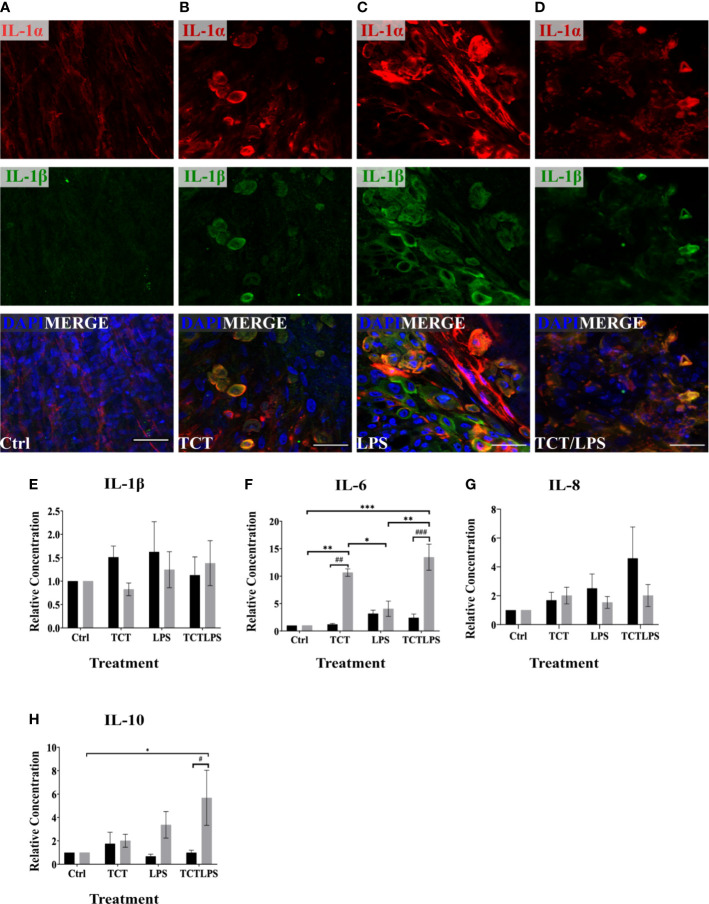
TCT/LPS modulate inflammatory cytokine expression in human airway mucosa models. The hTBM were treated with 3µM TCT, 100 ng LPS and a combination of TCT/LPS at the same concentrations for 24 h. The hTBM were fixed on the cell crowns with 4% PFA and immunostained for the proinflammatory cytokines IL-1α (red) and IL-1β (green). Z-stacks were made with a confocal microscope from the top of the epithelial layer to the basal membrane and reconstructed with FIJI. The z-projections for control **(A)** treated hTBM as well as TCT **(B)**, LPS **(C)** and TCT/LPS **(D)** are displayed in the image. The cell culture supernatants from the apical and basal compartments were analyzed for secreted IL-1β **(E)**, IL-6 **(F)**, IL-8 **(G)**, IL-10 **(H)** post toxin treatment. The concentrations (pg/ml) from the different treatment groups was normalized to the mock treated hTBM to eliminate donor variations and expressed as relative concentration. Data represents mean ± SEM of at 3 independent experiments performed in duplicate (N=6). * shows statistical significance between the different treatment groups (*p<0.05, **p<0.01, ***p<0.001) and *#* indicates statistical difference between apical and basal compartment of the same treatment groups (^#^p<0.05, ^##^p<0.01, ^###^p<0.005). All comparisons that did not show statistical significance do not carry any annotations on them. Scale bar 50µm.

The pathology of TCT in the tracheobronchial mucosa models is reported to be linked to the expression of IL-1. The internalization of TCT has been shown to be mediated by the SLC46A2 transmembrane receptor (Kim et al., 2015) which is expressed by the hTBM (**Supplementary Figure 3**). The internalized TCT interacts with cytoplasmic NOD receptors to induce the expression of proinflammatory cytokine through NF-κB activation (Uehara et al., 2005; Ratner et al., 2007). The addition of exogenous IL-1 to hamster tracheal explants has also been shown to produce similar effects as TCT (Heiss et al., 1993; Kim et al., 2015). IL-1 is therefore implicated as an intermediary in the disruptive effect of TCT on the respiratory mucosa in hamster tracheal cells. It, however, remains to be shown if TCT is able to induce IL-1 independently *in vivo*. In order to assess the induction of IL-1 expression by TCT, the hTBM were fixed with 4%PFA after the 24 h incubation and immunostained with antibodies specific for IL-1α and IL-1β. As shown in **Figures 5A–D**, immunostaining showed a clear increase in the expression of IL-1α and IL-1β as can be observed after incubation with TCT or LPS alone and in combination. Further analysis of the inflammatory cytokine induction at the mRNA level showed a 2-fold increase in *IL-1α* gene expression in hTBM which were incubated with TCT alone and TCT/LPS (**Supplementary Figure 4A**). Interestingly, incubation of the models with TCT/LPS resulted in a reduced expression of *IL-1β* after 24 h (**Supplementary Figure 4B**). Also, while TCT alone induced a significant decline in the pro-inflammatory cytokine *IL-6* expression, the combination of TCT/LPS caused its upregulation. Since these data on cytokine expression in part were not statistically significant, we further assessed the role of toxin mediated regulation of cytokine secretion into the cellular supernatants from apical and basal compartments. For this, we collected these supernatants and analyzed them using a cytometric bead array. As shown in **Figure 5E**, incubation of the hTBM with either toxins alone or in combination did not result in a significantly higher IL-1β secretion into the supernatant after 24 h. However, our results show significantly higher relative concentration of IL-6 in the supernatants from toxin treated compared to mock treated hTBM especially in the basal compartment (**Figure 5F**). Incubation of the hTBM with TCT/LPS also induced a very significant release of IL-6 into the basal compartment compared to mock controls and LPS alone (**Figure 5F**). TCT alone resulted in a significantly higher IL6 secretion into the basal compartment compared to control and LPS treated models. Furthermore, we also observed a significant increase in the expression of the inhibitory IL-10 in TCT/LPS treated models (**Figure 5H**). While IL-6 and IL-10 showed a more basally directed secretion, IL-1β and IL-8 showed a more apically directed secretion (**Figure 5G**). The data indicate that TCT and LPS may modulate the expression of inflammatory cytokines besides the IL-1.

## Discussion

The pathological effects of tracheal cytotoxin (TCT) of *Bordetella pertussis* have been extensively documented using hamster tracheal explants as a model (Goldman et al., 1982; Goldman & Herwaldt, 1985; Flak et al., 2000). The role of TCT in the pathophysiology of *B. pertussis* is however still a matter of debate mainly due to the scant human data. Thus, the aim of this study was to assess the potential contribution of TCT to cytopathology of infection in a human *in vitro* airway model developed from primary tracheobronchial epithelial cells and fibroblasts (Steinke et al., 2014; Schweinlin et al., 2017). Compared to similar models developed from cell lines, this hTBM consistently shows high *in vivo – in vitro* correlation producing functional cilia which cover at least 60% of the tissue surface (Steinke et al., 2014; Lodes et al., 2020). Furthermore, these models consistently show pseudostratification of the epithelium, produce mucus and express relevant airway epithelia cell markers (**Supplementary Figure 1**).

A major pathophysiology of the airway provoked by *B. pertussis* infection is the necrosis and blebbing of the epithelium which results in denuding of ciliated cells as observed in autopsies of fatal cases (Paddock et al., 2008). Ultrastructural analysis of the hTBM after 24 h of TCT incubation confirmed earlier results obtained with hamster tissue which showed that TCT and/or LPS can cause this phenotype and that TCT and LPS may even act synergistically, since in the presence of both compounds tissue disruption was even more pronounced (Cookson et al., 1989; Heiss et al., 1993; Luker et al., 1995). We observed cellular debris and rupture of the cellular membrane typical of necrotic cells (**Figure 1B**). However, an interesting difference between the hamster model and the hTBM was found in the fact that the cytotoxic effect of TCT was not restricted to ciliated cells as described for the hamster model. Instead, non-ciliated cells such as goblet cells equally experienced cytotoxicity and blebbing (**Figure 1**). Similarly, using human nasal biopsies cytoplasmic blebbing and cell extrusions were observed equally in ciliated and unciliated cells after TCT treatment (Wilson et al., 1991). The effect of the toxin may however be more pronounced on the ciliated cells due to their higher energy requirements to maintain ciliary beating. These observations may thus indicate species specific differences in response to the toxin. Moreover, the blebbing and cellular extrusion resulted in the disruption of the tight junction organization and function which is located apically in the hTBM.

The disruptive effect of TCT was reported to be mediated by cytokine-dependent release of nitric oxide into the culture supernatant (Heiss et al., 1993; Flak & Goldman, 1999; Flak et al., 2000; Abramson et al., 2001). We observed an increased expression of iNOS (**Figure 4**) in toxin treated hTBM as well as consistent increases in NO dissolved in the supernatants of TCT and TCT/LPS treated hTBM although the differences were statistically not significant (**Supplementary Figure 2A**). It is likely that the use of primary cells from different donors for the generation of the tissue models may be a major reason for statistical insignificance due to strong variations in donor-to-donor response. Thus, the role of NO in tissue destruction after intoxication of hTBM remains inconclusive and requires further studies. Nevertheless, the observed combined increases in iNOS expression and NO concentration point to a similar role of iNOS produced NO in human airway cells as previously described using hamster tracheal rings (Heiss et al., 1993; Flak & Goldman, 1999; Flak et al., 2000), where mucus secreting goblet cells have been reported to be the source of the iNOS-dependent and toxin induced NO released (Flak & Goldman, 1999).

Because previous work indicated a link between TCT intoxication and ciliostasis, which can be defined as the slowing or complete halting of ciliary activity, in hamster tracheal explants (Goldman & Cookson, 1988; Luker et al., 1993), we investigated whether TCT interferes with cilia activity in the hTBM. The data indicate only a marginal effect of TCT on the ciliary beat frequency which after 24 h was still above the reported physiological levels of approximately 9 Hz (Katz et al., 1987; Veale et al., 1993; Sanderson & Dirksen, 1995; Sedaghat et al., 2016). However, it should be noted that only intact ciliated cells with beating cilia were included in the assessment of CBF. Furthermore, additional parameters may interfere with CBF, e.g., increased NO concentrations had previously been shown to upregulate the ciliary beat frequency (Jain et al., 1993). Since our preliminary data indicate that TCT induces NO production, this increase in NO concentration may contribute to the quite high CBF recorded after TCT intoxication of the hTBM despite the observed tissue disruption. Thus, in the hTBM, TCT and LPS seem not to directly affect ciliary function but mainly affect viability of epithelial cells and increase viscous mucus production. The viscous mucus subsequently traps cell debris and thus inhibits the clearance mechanisms as shown in **Supplementary Video 16**. It will be important to further investigate the consequences of increased mucus production stimulated by the toxins on particle transport. These results are in line with previous observations by Wilson et al. (1991) who reported that using human nasal biopsies culture filtrates of *B. pertussis* or purified TCT did not affect CBF significantly at least after 4 h of incubation but a loss of ciliation by killing and extrusion of ciliated cells. In contrast, Anderton et al. (2004) using dog trachea organ culture observed a significant early effect on the ciliary-mediated clearance of beads after infection with virulent *B. bronchiseptica* and suggested ciliostasis to be caused while tissue damage appeared to be quite minimal.

The ability of TCT to induce cell cytotoxicity was reported to be linked to the induction of IL-1 in airway epithelial cells in the hamster model (Dinarello & Krueger, 1986; Heiss et al., 1993; Flak & Goldman, 1996). IL-1 has previously been demonstrated to exert cytotoxic effects and to inhibit cell proliferation (Heiss et al., 1993). A correlation between IL-1 stimulation and the pyrogenic effect of muramyl peptides has also been reported previously (Dinarello et al., 1978; Dinarello & Krueger, 1986; Dinarello, 1989). TCT is a muramyl peptide which is known to induce the expression of cytokines through cytoplasmic NOD-like receptor protein 1 (NOD1). NOD-like receptors are known to induce inflammatory responses through NF-κB activation (Kobayashi et al., 2002; van Heel et al., 2005; Strober et al., 2006; Kufer, 2008; Moreno et al., 2010; Paik et al., 2017). However, Magalhaes et al. (2005) reported that human NOD1 appears to be less sensitive to TCT compared to mouse NOD1 and suggested there may be a yet unidentified receptor for TCT detection in specific human cells such as the airway epithelial cells. On the other hand, Paik et al. (2017) recently provided evidence that NOD1 should in fact be a sensitive TCT receptor in human airway cells. However, NOD1 signaling is dependent on efficient TCT delivery by the SLC46A2 transporter. We show here that the SLC46A2 transporter is expressed in the hTBM and hence NOD1 mediated signaling as a response to TCT is likely to be relevant (**Supplementary Figure 3**). LPS induction of inflammation is mediated by the Toll-like receptor 4 (Reviewed in Park & Lee, 2013). Consequently, these two pathways converge to sustain the activation and subsequent nuclear localization of NF-κB resulting in a sustained inflammatory reaction. Additionally, IL-1α may act as a transcription factor in the nucleus and may promote the expression of chemokines such as IL-8 or bind to its membrane receptor to initiate inflammatory signal transduction (Werman et al., 2004). IL-1β has also been demonstrated to induce migration and differentiation in airway epithelial cells after airway mucosa damage (White et al., 2008). The low expression of IL-1 observed in our experiments after 24 h is likely the result of its early induction and secretion during inflammation and its short half-life (Turner et al., 1989; Werman et al., 2004). The potentially reduced sensitivity of the human NOD1 which mediates the induction of inflammatory cytokines by muramyl peptides as reported by Magalhaes et al. (2005) may also contribute to the low IL-1 expression we observed. Furthermore, IL-1 promotes the expression of IL-6, another cytokine that was significantly upregulated by TCT and TCT/LPS (Mizutani et al., 1989).

IL-6 is known to promote antibody production and CD4 T cell activation (Mizutani et al., 1989; Ben-Baruch et al., 1995; Higgs et al., 2012). It has however recently been reported to promote the differentiation of basal cells into multi-ciliated cells through STAT3 induction of *Foxj1* (Tadokoro et al., 2014). The increased release of these cytokines into the basal compartment is indicative of its function in the recruitment of T-cells from the submucosal regions. IL-8 is a potent chemoattractant and stimulator of neutrophil accumulation in the respiratory system upon infection (Kunkel et al., 1991; Teran et al., 1997) while IL-10 serves to inhibit inflammation, thus reducing the damaging effect of the tissues due to inflammation (Cassatella et al., 1993). Recruited neutrophils at the sites of inflammation secrete more IL-8 thus sustaining inflammatory reaction (Kunkel et al., 1991; Cassatella et al., 1992; Park et al., 1998). Despite our results showing about a 5-fold IL-10 secretion in TCT/LPS treated hTBM compared to controls, the actual concentration was 4.10 pg/ml. This concentration is massively dwarfed by the concentration of IL-6 and IL-8 secreted into the supernatants (**Supplementary Table 3**). TCT and LPS may therefore collaborate to upregulate inflammation while downregulating IL-10. Hence, TCT seems to upregulate the expression of the proinflammatory cytokines while downregulating anti-inflammatory IL-10. Inflammatory cytokines present at sites of inflammation in the airway can alter the mucociliary apparatus thus affecting the mucociliary clearance mechanism. Inflammatory cytokines have also been reported to induce tight junction disassembly in epithelial cells by altering actin structure and downregulating tight junction proteins (Reviewed in Capaldo & Nusrat, 2009). In the future, experiments are planned with *B. pertussis* lipooligosaccharide (LOS) in order to unravel potential differences in their activities in comparison to data shown here using *E. coli* LPS. However, despite the differences in the structure of LOS and LPS, they have been shown to have very similar biological activity in terms of lethal toxicity in animal studies (Watanabe et al., 1990; Flak et al., 2000). Furthermore, the role of inflammatory cytokines in the host cell response to TCT/LPS needs to be investigated further.

Taken together, the results suggest that TCT and LPS play a role in the colonization of the human airway epithelia by *Bordetella pertussis*. The disruption of tight junction organization and function coupled with necrosis and extrusion of epithelial cells is essential for accessing the submucosal layers. TCT induced tissue destruction may occur early during *Bordetella* infection to aid bacterial colonization and metabolite acquisition, thus paving the way for other toxins, such as the adenylate cyclase toxin which is known to affect cells mainly from the basolateral side, to induce further cytopathology. In addition, the loss of ciliated cells and hyper-mucus production impairs mucociliary clearance mechanism suggesting a contribution of TCT in pathology and possibly the cough mechanism that characterizes whooping cough.

## Data Availability Statement

The original contributions presented in the study are included in the article/**Supplementary Material**. Further inquiries can be directed to the corresponding author.

## Ethics Statement

Informed consent was obtained from patients before surgery and the studies were approved by the institutional ethics committees on human research of the Otto-von-Guericke University Magdeburg (vote 163/17) and Julius-Maximilians-University Würzburg (vote 179/17), respectively.

## Author Contributions

RG and MS designed the experiments. DK conducted the experiments. NL analyzed ciliary beating frequency. HO performed histology. WG and TW provided the material. DK, MS, and RG analyzed the results and wrote the manuscript. All authors contributed to the article and approved the submitted version.

## Funding

Funding for this work was provided by the Deutsche Forschungsgemeinschaft (DFG) GRK2157 3D Tissue Models for Studying Microbial Infection by Human Pathogens to RG and MS. This publication was funded by the German Research Foundation (DFG) and the University of Wuerzburg under the Open Access Publishing funding program.

## Conflict of Interest

The authors declare that the research was conducted in the absence of any commercial or financial relationships that could be construed as a potential conflict of interest.

## Supplementary Material

Supplementary Figure 1 Characterization of engineered airway mucosa models. Engineered airway mucosa models express markers typical of the airway epithelia such as cytokeratin 14 (CK14) for epithelial cells and β-tubulin (B-tub) for cilia **(A),** respiratory mucosa specific cytokeratin 18 (CK18) **(B)** and cytokeratin 5 (CK5) for basal cells **(D)**. The tissue models also possess goblet cells which produce mucus containing Muc5AC **(C)** and Muc5B **(B).** The presence of fibroblast in the scaffold was confirmed by immunostaining with vimentin (VIM) **(C)**.

Supplementary Figure 2 Induction of nitric oxide. Models were incubated with the different toxins as previously described. One set of hTBM were ventilated by taking off the plate cover under a flow chamber to simulate airflow. Nitric oxide in the supernatant in the apical and basal compartments assessed using the Griess reagent. **(A)** Nitric oxide quantified in the supernatants from apical and basal compartments of the hTBM after toxin incubation. **(B)** Ventilation does not significantly affect the NO accumulation in media. Bar chart displays the mean±SEM of three independent experiments.

Supplementary Figure 3 Immunofluorescence images of the human tracheobronchial mucosa models showing the expression of the SLC46A2 membrane receptors. 10 µm sections of formalin fixed, and paraffin embedded sections were decorated with anti-SLC46A2 antibody, counterstained with fluorescently conjugated secondary antibody and imaged on fluorescent microscope.

Supplementary Figure 4 The relative gene expression of inflammatory cytokines; *IL-1α* **(A)** *IL-1β* **(B)** *IL-6* **(C)** and *IL-10* **(D)**, were analyzed after incubating the hTBM with TCT (3µM), LPS (100 ng/ml) and TCT/LPS from the apical side for 24 h. *Gapdh* was used as the reference gene. Bars represents mean ± SEM of at least 3 independent experiments performed in duplicate (N=6). A Tukey test did not show significant statistical differences between the different treatment groups for *IL-1α*, *IL-1β* and *IL-10.* TCT*/*LPS induced significant expression of *IL-6* compared to either toxin alone (^ns^p>0.05, *p<0.05, **p<0.01).

Supplementary Figure 5 Particle movement line graphs derived from highspeed videos of particle transport on the hTBM. High speed videos of the movement of Dynabead™ Protein G particles were recorded before **(A)** and after **(B)** treatment of the hTBM. The translocation of these particles over 100 frames (approximately 1 sec) were assessed using Image-Pro video analyses software. Colored lines with arrowheads show direction and path of tracked particles over the 100 frames.

Supplementary Table 1 List of primary antibodies used in this study.

Supplementary Table 2 List and sequence of primers used for quantitative RT-PCR.

Supplementary Table 3 Mean concentrations and standard deviations (pg/ml) of inflammatory cytokines quantified from the cell culture supernatant using the cytometric bead array kit.

Supplementary Video 1 Movie of beating cilia recorded before mock treating hTBM with fresh mixed media. Video was recorded for 10 secs at a frame rate of 100 fps.

Supplementary Video 2 Movie of beating cilia of the hTBM recorded before the addition of TCT to the apical side. Video was recorded for 10 secs at a frame rate of 100 fps.

Supplementary Video 3 Movie of beating cilia of the hTBM recorded before addition of LPS to the apical side. Video was recorded for 10 secs at a frame rate of 100 fps.

Supplementary Video 4 Movie of beating cilia of the hTBM recorded before the addition of TCT/LPS to the apical side. Video was recorded for 10 secs at a frame rate of 100 fps.

Supplementary Video 5 Movie showing the distribution of beating cilia after mock treating hTBM for 24 h with fresh mixed media from the apical side. Video was recorded for 10 secs at a frame rate of 100 fps.

Supplementary Video 6 Movie showing distribution of beating cilia after treating hTBM with 3µM TCT for 24 h from the apical side. Video was recorded for 10 secs at a frame rate of 100 fps.

Supplementary Video 7 Movie showing distribution of beating cilia after treating hTBM for with 100 ng/ml LPS for 24 h from the apical side. Video was recorded for 10 secs at a frame rate of 100 fps.

Supplementary Video 8 Movie showing the distribution of beating cilia and hyper mucus production in hTBM incubated with TCT/LPS for 24 h from the apical side. Video was recorded for 10 secs at a frame rate of 100 fps.

Supplementary Presentation 1 Dynabeads™ Protein G particles (black moving spots) are translocated by the beating cilia in hTBM. The Dynabeads™ Protein G particles (particles) were mixed into fresh mixed media to a final concentration of 15µg/ml and pipetted onto hTBM pinned on Bioptech^®^ Delta T dishes. 15 s long videos of the transport of the particles across the surface of the hTBMs were recorded at a frame rate of 200 fps before and 24 h after the addition of TCT, LPS, TCT/LPS or fresh mixed media (Mock treatment). Supplementary videos 9, 10, 11 and 12 are videos of the transport of the particles on the hTBM before treatment. Supplementary video 13, 14, 15, 16 are videos of the particles on the hTBM recorded 24 h after the addition of mixed media, TCT, LPS and TCT/LPS respectively to the apical side.

Supplementary Video 9 Video recording of particle transport of hTBM prior to mock treatment.

Supplementary Video 10 Video recording of particle transport of hTBM prior to addition of 3µM TCT to the apical side.

Supplementary Video 11 Movie showing particle transport of hTBM prior to addition of 100 ng/ml LPS to the apical side.

Supplementary Video 12 Movie showing particle transport of hTBM prior to addition of TCT/LPS to the apical side.

Supplementary Video 13 Movie showing particle transport of 24-h mock treated hTBM.

Supplementary Video 14 Movie showing particle transport of 24-h TCT treated hTBM.

Supplementary Video 15 Movie showing particle transport of 24-h LPS treated hTBM.

Supplementary Video 16 Movie of 24-h TCT/LPS treated hTBM showing particles trapped in mucus and oscillating at areas with beating cilia.
