# Supplementary figures and images for: Activity of Tracheal Cytotoxin of Bordetella pertussis in a Human Tracheobronchial 3D Tissue Model
Source: Front Cell Infect Microbiol. 2021 Jan 19;10:614994. doi: 10.3389/fcimb.2020.614994 (PMC7873972; doi:10.3389/fcimb.2020.614994)

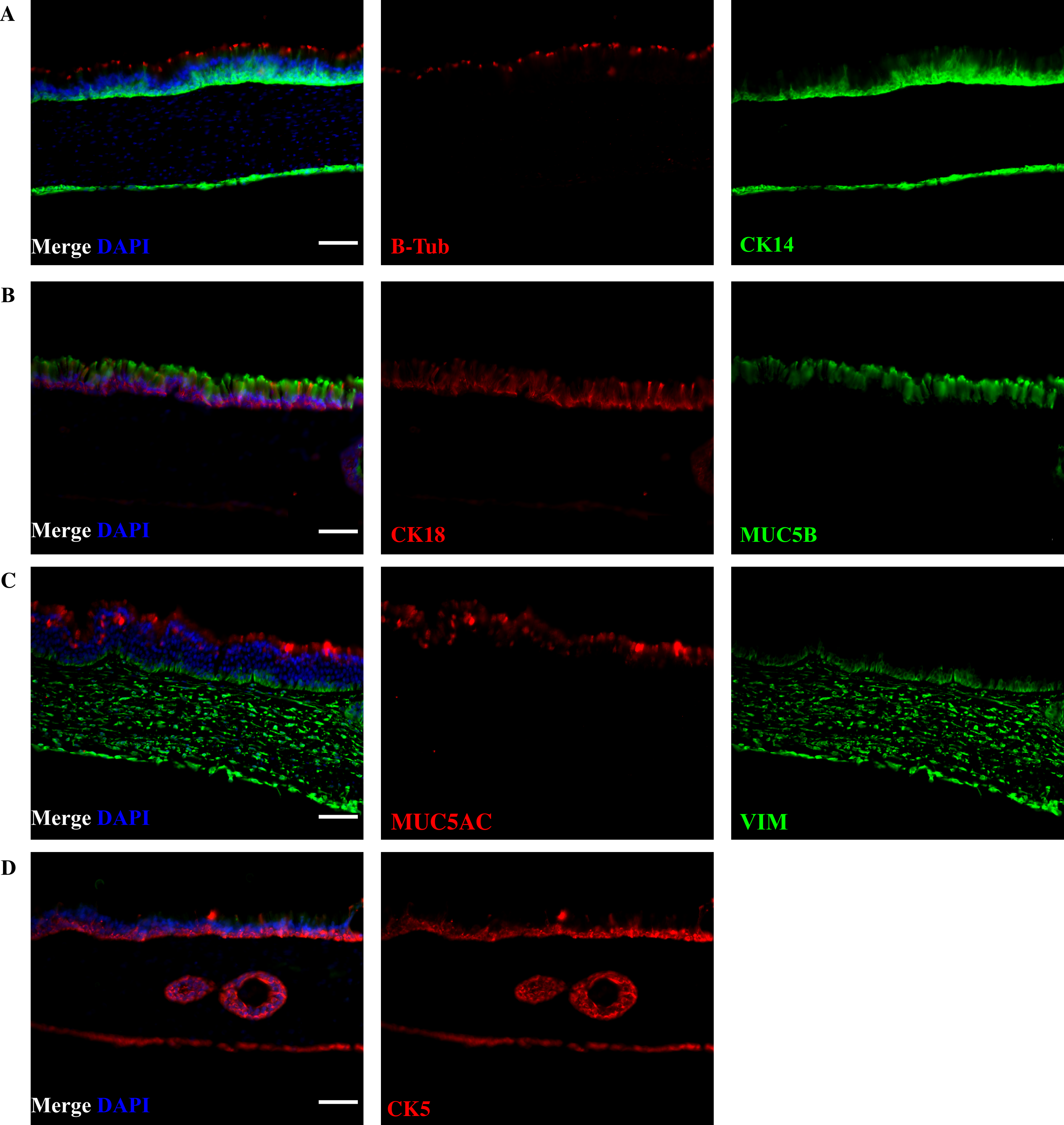

Supplement: Supplementary Figure 1 — Characterization of engineered airway mucosa models. Engineered airway mucosa models express markers typical of the airway epithelia such as cytokeratin 14 (CK14) for epithelial cells and β-tubulin (B-tub) for cilia (A), respiratory mucosa specific cytokeratin 18 (CK18) (B) and cytokeratin 5 (CK5) for basal cells (D). The tissue models also possess goblet cells which produce mucus containing Muc5AC (C) and Muc5B (B). The presence of fibroblast in the scaffold was confirmed by immunostaining with vimentin (VIM) (C). [file Image_1.tiff]

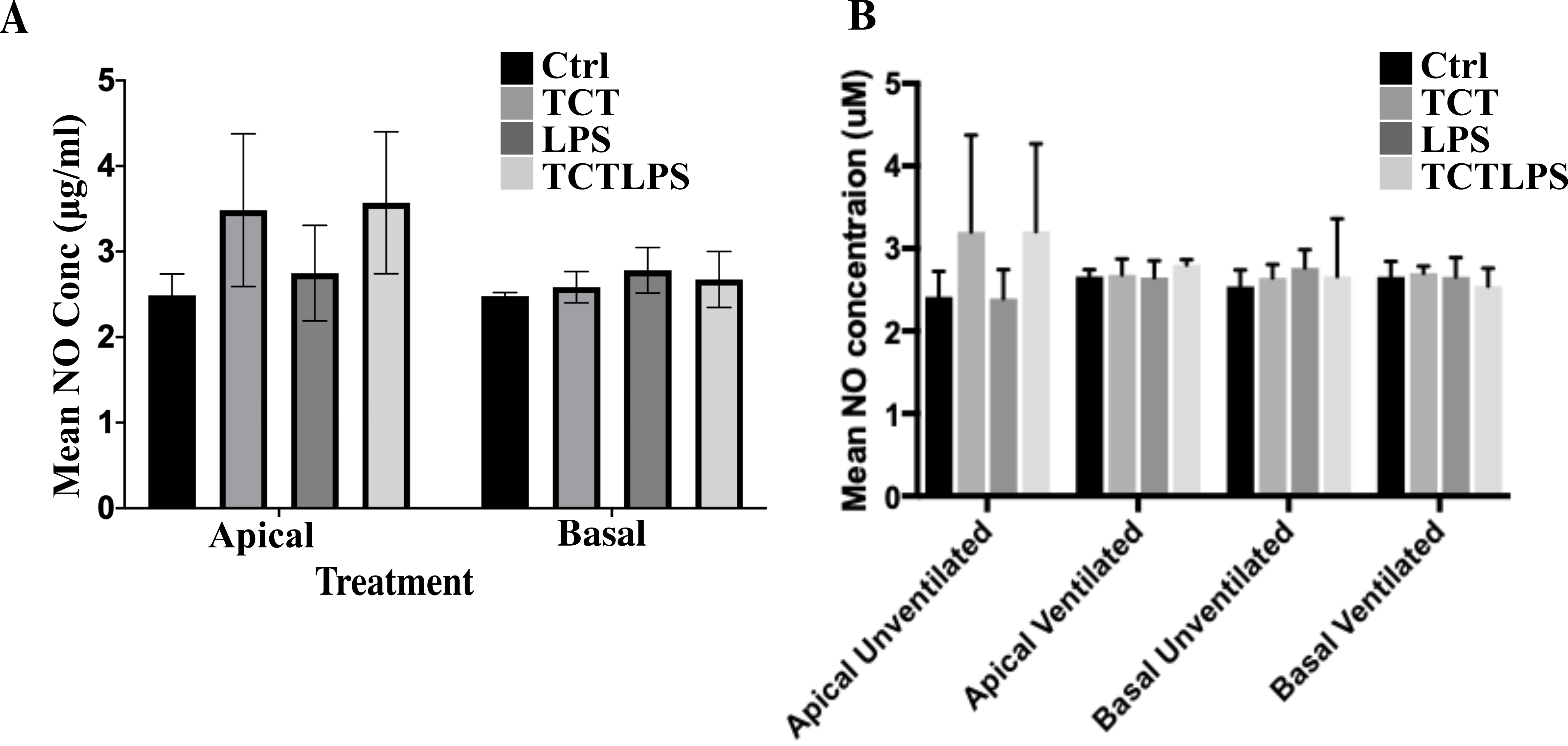

Supplement: Supplementary Figure 2 — Induction of nitric oxide. Models were incubated with the different toxins as previously described. One set of hTBM were ventilated by taking off the plate cover under a flow chamber to simulate airflow. Nitric oxide in the supernatant in the apical and basal compartments assessed using the Griess reagent. (A) Nitric oxide quantified in the supernatants from apical and basal compartments of the hTBM after toxin incubation. (B) Ventilation does not significantly affect the NO accumulation in media. Bar chart displays the mean±SEM of three independent experiments. [file Image_2.tiff]

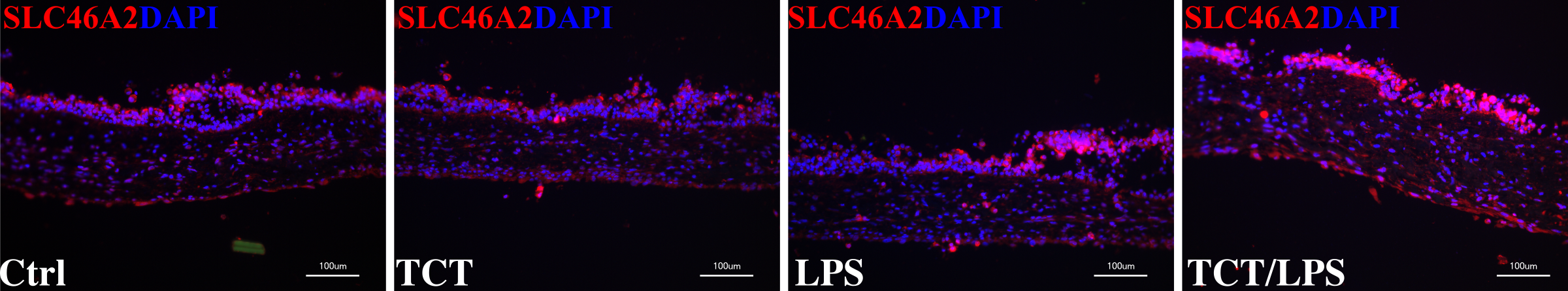

Supplement: Supplementary Figure 3 — Immunofluorescence images of the human tracheobronchial mucosa models showing the expression of the SLC46A2 membrane receptors. 10 µm sections of formalin fixed, and paraffin embedded sections were decorated with anti-SLC46A2 antibody, counterstained with fluorescently conjugated secondary antibody and imaged on fluorescent microscope. [file Image_3.tiff]

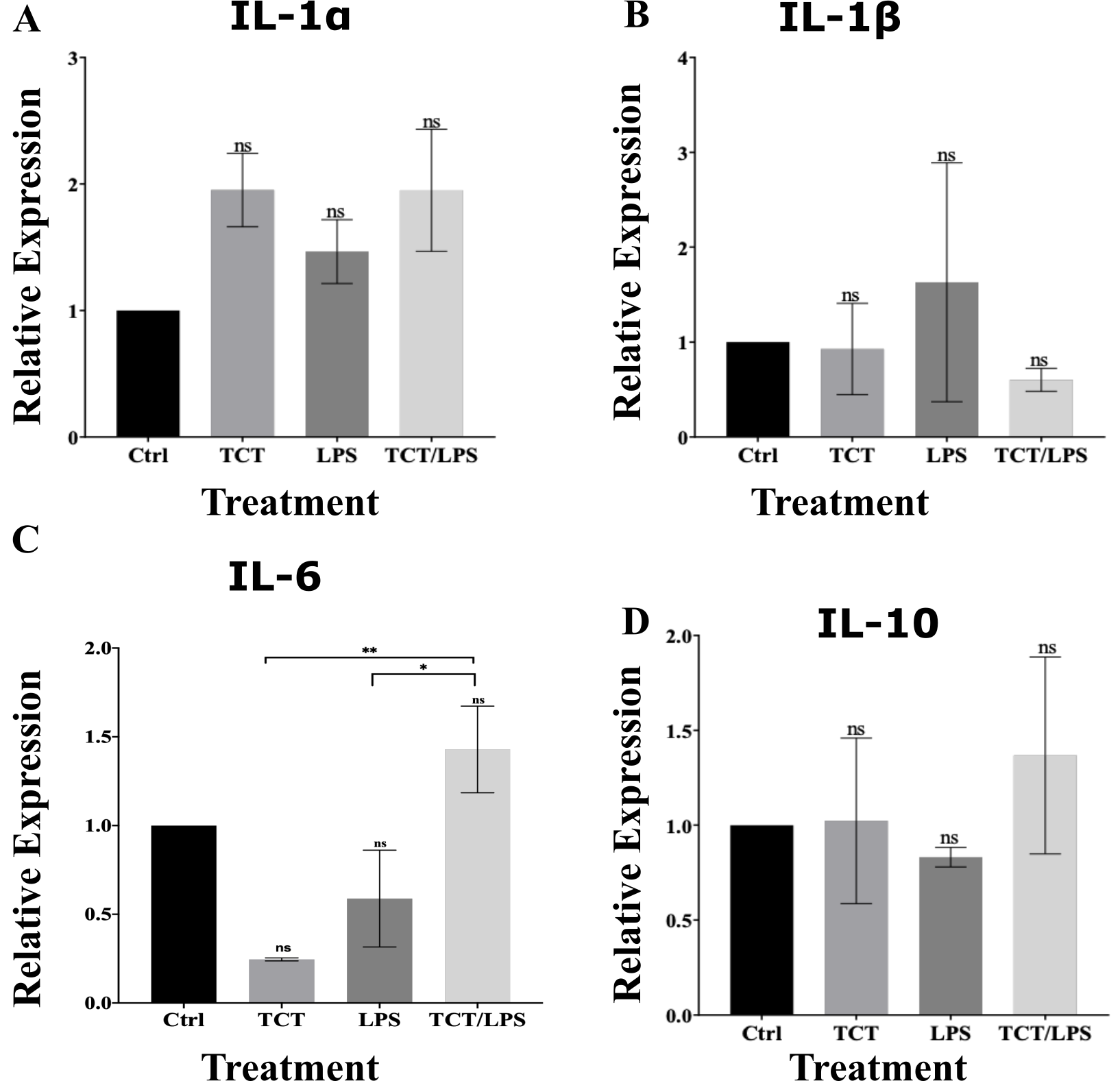

Supplement: Supplementary Figure 4 — The relative gene expression of inflammatory cytokines; IL-1α (A) IL-1β (B) IL-6 (C) and IL-10 (D), were analyzed after incubating the hTBM with TCT (3µM), LPS (100 ng/ml) and TCT/LPS from the apical side for 24 h. Gapdh was used as the reference gene. Bars represents mean ± SEM of at least 3 independent experiments performed in duplicate (N=6). A Tukey test did not show significant statistical differences between the different treatment groups for IL-1α, IL-1β and IL-10. TCT/LPS induced significant expression of IL-6 compared to either toxin alone (nsp>0.05, *p<0.05, **p<0.01). [file Image_4.tiff]

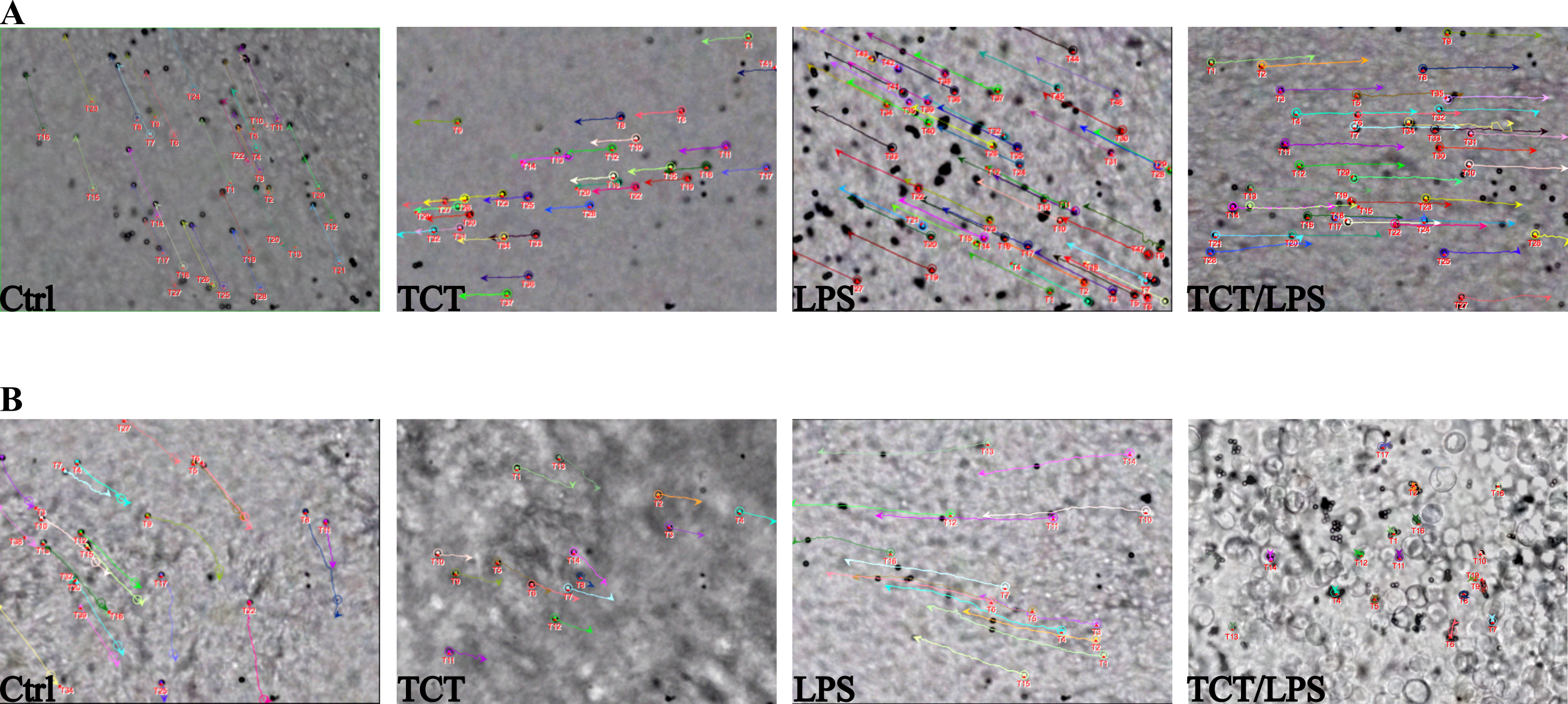

Supplement: Supplementary Figure 5 — Particle movement line graphs derived from highspeed videos of particle transport on the hTBM. High speed videos of the movement of Dynabead™ Protein G particles were recorded before (A) and after (B) treatment of the hTBM. The translocation of these particles over 100 frames (approximately 1 sec) were assessed using Image-Pro video analyses software. Colored lines with arrowheads show direction and path of tracked particles over the 100 frames. [file Image_5.tiff]
