# Supplementary material for: Activity of Tracheal Cytotoxin of Bordetella pertussis in a Human Tracheobronchial 3D Tissue Model
Source: Front Cell Infect Microbiol. 2021 Jan 19;10:614994. doi: 10.3389/fcimb.2020.614994 (PMC7873972; doi:10.3389/fcimb.2020.614994)
Supplement: Supplementary Table 1 — List of primary antibodies used in this study. [file Table_1.docx]

**Supplementary Table 1**

| **Target** | **Host** | **Dilution** | **Company (catalog)** |
| --- | --- | --- | --- |
| IL1-α | Rabbit polyclonal | 1:100 | Abcam (Ab7632) |
| IL-1β | Mouse monoclonal | 1:100 | Thermo Scientific (M421B) |
| SLC46A2 | Rabbit Polyclonal | 1:500 | Thermo scientific (PA5-31389) |
| iNOS | Rabbit polyclonal | 1:500 | Thermo Scientific (PA3-030A) |
| Cytokeratin 18 | Mouse monoclonal | 1:100 | Dako (M7010) |
| Cytokeratin 14 | Rabbit polyclonal | 1:1000 | Sigma (HPA023040) |
| Cytokeratin 5/6 | Mouse monoclonal | 1:200 | Dako (M7237) |
| Muc5AC | Mouse monoclonal | 1:100 | Sigma (HPA008246 |
| Muc5B | Rabbit polyclonal | 1:100 | Thermo Scientific (MA1-38223) |
| β-tubulin | Mouse monoclonal | 1:1000 | Sigma (T8328) |
| ZO-1 | Rabbit polyclonal | 1:1000 | Proteintech (21773-1-AP) |
| E-cadherin | Mouse monoclonal | 1:100 | BD Biosciences (610181) |
| Vimentin | Rabbit monoclonal | 1:1000 | Abcam (Ab92547) |
