# Supplementary material for: Activity of Tracheal Cytotoxin of Bordetella pertussis in a Human Tracheobronchial 3D Tissue Model
Source: Front Cell Infect Microbiol. 2021 Jan 19;10:614994. doi: 10.3389/fcimb.2020.614994 (PMC7873972; doi:10.3389/fcimb.2020.614994)
Supplement: Supplementary Table 2 — List and sequence of primers used for quantitative RT-PCR. [file Table_2.docx]

**Supplementary table 2**

| *IL-1a* | sense | CTTAGTGCCGTGAGTTTCCC |
| --- | --- | --- |
| *IL-1a* | antisense | TGTGACTGCCCAAGATGAAG |
| *IL-1 b* | sense | AAGCCCTTGCTGTAGTGGTG |
| *IL- 1 b* | antisense | GAAGCTGATGGCCCTAAACA |
| *IL-6* | sense | CATTTGTGGTTGGGTCAGG |
| *IL-6* | antisense | AGTGAGGAACAAGCCAGAGC |
| *IL 10* | sense | CTCATGGCTTTGTAGATGCCT |
| *IL 10* | antisense | GCTGTCATCGATTTCTTCCC |
| *GAPDH* | sense | TGACGCTGGGGCTGGCATTG |
| *GAPDH* | antisense | GCTCTTGCTGGGGCTGGTGG |
