# Supplementary material for: Activity of Tracheal Cytotoxin of Bordetella pertussis in a Human Tracheobronchial 3D Tissue Model
Source: Front Cell Infect Microbiol. 2021 Jan 19;10:614994. doi: 10.3389/fcimb.2020.614994 (PMC7873972; doi:10.3389/fcimb.2020.614994)
Supplement: Supplementary Table 3 — Mean concentrations and standard deviations (pg/ml) of inflammatory cytokines quantified from the cell culture supernatant using the cytometric bead array kit. [file Table_3.docx]

**Supplementary table 3**

|  | **Apical** | **Basal** |
| --- | --- | --- |
|  | **Mean Conc ± SD (pg/ml)** | **Mean Conc ± SD (pg/ml)** |
|  | **IL-1β(n=5)** | |
| **Control** | 7.86 ± 5.94 | 11.30 ± 4.41 |
| **TCT** | 10.32 ± 7.76 | 10.10 ± 5.58 |
| **LPS** | 11.43 ±1 0.23 | 11.26 ± 5.41 |
| **TCT/LPS** | 5.81 ± 3.32 | 16.06 ± 12.99 |
|  | **IL-6 (n=3)** | |
| **Control** | 3097.53 ± 2312.51 | 5649.74 ± 6505.70 |
| **TCT** | 3306.83 ± 1929.08 | 60028.79 ± 69309.55 |
| **LPS** | 10807.25 ± 8498.14 | 32684.65 ± 47869.68 |
| **TCT/LPS** | 7418.80 ± 5123.85 | 63969.40 ± 62382.40 |
|  | **IL-8 (n=6)** | |
| **Control** | 18730.18 ± 14789.57 | 92656.91 ± 93830.22 |
| **TCT** | 21981.96 ± 15533.16 | 144289.31 ± 96409.01 |
| **LPS** | 46463.72 ± 32639.59 | 120568.57 ± 87677.80 |
| **TCT/LPS** | 42435.49 ± 12790.09 | 125748.82 ± 81826.63 |
|  | **IL-10 (n=6)** | |
| **Control** | 0.54 ± 0.71 | 2.75 ± 3.44 |
| **TCT** | 0.69 ± 0.80 | 2.47 ± 2.65 |
| **LPS** | 0.44 ± 0.71 | 4.87 ± 4.35 |
| **TCT/LPS** | 0.54 ± 0.73 | 4.10 ± 3.62 |
